# Guizhi Fuling Capsule Exhibits Antidysmenorrhea Activity by Inhibition of Cyclooxygenase Activity

**DOI:** 10.1155/2020/8607931

**Published:** 2020-05-23

**Authors:** Weiran Zheng, Meng Li, Yongxiang Wang, Baojie Lv, Xinzhuang Zhang, Lilan Chen, Kejin Zhu, Zhenzhong Wang, Baoxin Li, Wei Xiao

**Affiliations:** ^1^Harbin Medical University, Harbin, Heilongjiang 150081, China; ^2^State Key Laboratory of New-Tech for Chinese Medicine Pharmaceutical Process, Jiangsu Kanion Pharmaceutical Co., Ltd., Lianyungang, Jiangsu 222001, China; ^3^Jiangsu Key Laboratory of Molecular Medicine, Medical School, Nanjing University, Nanjing, Jiangsu 210093, China

## Abstract

Guizhi Fuling capsule (GZFLc) is a modern preparation from traditional Chinese Medicine. Guizhi Fuling was first prescribed by Zhang Zhongjing almost two thousand years ago for the treatment of primary dysmenorrhea. It has also been used to treat uterine fibroids, dysfunctional uterine bleeding, and endometriosis. Although effective against dysmenorrhea clinically, there are limited information on the mechanism of its action. The major components responsible for the activity are not well defined. The aim of this study has been to elucidate a mechanism that may facilitate the development of a bioactivity-based assay for quality control during drug formulation and manufacturing. Using an oxytocin-induced mouse dysmenorrhea model, we showed that oral administration of GZFLc at 150 and 300 mg/kg, dosages relevant to clinic usages, significantly suppressed oxytocin-induced writhing response. The antidysmenorrhea effect was also demonstrated by a rotarod assay. We showed that GZFLc treatment significantly prolonged the hanging time of mice on the rotating rod. Histological studies showed that GZFLc treatment reduced lamina propria edema, while no effect on COX2 expression was detected. GZFLc instead exhibited direct inhibitory effect against COX2, a critical enzyme that catalyzes arachidonic acid conversion to prostaglandins. By HPLC profiling, we showed that paeoniflorin, paeonol, and cinnamaldehyde are the major components from the corresponding plants. At 5 and 10 mg/kg, both paeoniflorin and paeonol were active against induced dysmenorrhea. The study not only links GZFLc antidysmenorrhea activity to COX2 inhibition but also uncovers a mechanism of action by which an assay can be developed for bioefficacy evaluation of GZFLc.

## 1. Introduction

Guizhi Fuling (GZFL) decoction is a classical formula first described in the Essential Prescriptions from the Golden Cabinet (Jingui Yaolue) by Zhang Zhongjing of the Han Dynasty (third century A. D.). The formula consists of five components, including Cassia Twig, Indian Bread, Peach Seed, White Peony Root, and Tree Peony Bark. “Cassia Twig” is the dried twigs of *Cinnamomum cassia* Presl (Fam. Lauraceae). The drug is collected in spring and summer, removed from leaf, and dried in the sun after being sliced. “Indian Bread” is the dried *sclerotium* of the fungus, *Poria cocos* (Schw.) Wolf (Fam. Polyporaceae). The drug is collected mostly in July to September, removed from the soil, piled up on the surface wet, and then spread on the surface to dry. “Peach Seed” is the dried ripe seed of *Prunus persica* (L.) Batsch or *Prunus davidiana* (Carr.) Franch. (Fam. Rosaceae). The fruit is collected when ripe. The seed is removed from sarcocarp and shell (endocarp) and dried in the sun. “White Peony Root” is the dried root of *Paeonia lactiflora* Pall.(Fam. Ranunculaceae). The drug is collected in summer or autumn, washed clean, removed from two ends and rootlet, either peeled after boiling in water or boiled after peeling, and dried in the sun. “Tree Peony Bark” is the dried root bark of *Paeonia suffruticosa* Andr. (Fam. Ranunculaceae). The root is collected in autumn and removed from rootlets and soil, and then the root bark is stripped off and dried in the sun. The five herbs work synergistically in invigorating blood circulation and unknotting blood stasis. The formula is capable of alleviating uterine discomfort or menstruation agitation, including amenorrhea and abdominal pains. A bibliometric review of modern literatures shows that GZFL is among the most frequently prescribed Chinese herbal formula for the treatment of endometriosis-related symptomatic discomfort [1, 2]. GZFL formula and several similar Wenjing decoctions have demonstrated equal or superior activities against symptoms of primary dysmenorrhea [3]. Given in combination with other drugs, it significantly reduces the volume of fibroids, compared to administration of only the allopathic drug.

Guizhi Fuling capsule (GZFLc) is a modern preparation for gynecological diseases. The drug can be used for uterine fibroids, dysfunctional uterine bleeding, and endometriosis and primary dysmenorrhea [4, 5]. Dysmenorrhea, also known as painful periods, or menstrual cramps, is a common gynecological illness among women of reproductive age. Over 60% of women experience primary dysmenorrhea of certain degree during menstruation periods in their early adulthood or adolescent age [6, 7]. It is believed that primary dysmenorrhea is caused by production of and subsequent action of prostaglandin F2*α* (PGF2*α*), oxytocin, and vasopressin [8, 9]. PGF2*α* release is significantly elevated in women with primary dysmenorrhea, causing the uterine musculature to contract and consequently pain. Nonsteroidal anti-inflammatory drugs (NSAIDs) are among the most effective therapies since they can inhibit cyclooxygenase (COX) activity and hence block prostaglandin production [10, 11]. Use of hormonal birth control may improve symptoms of primary dysmenorrhea, but they may induce amenorrhea [12]. As mentioned above, complementary and alternative medicines have also demonstrated effectiveness against primary dysmenorrhea [3, 13].

Previous studies have demonstrated that GZFLc possessed potent antidysmenorrhea activity [14]. The quality of a Chinese medicine preparation as complex as GZFLc depends not only on the quality of the herbal ingredients but also on the presence of the chemicals, which is generally determined by comparison to a fingerprint profile of HPLC analysis [15]. Therefore, an assay that determines the bioefficacy by mimicking the therapeutic scenario may be used to supplement or to replace the nonbiological activity-based assays for GZFLc quality control. We investigated whether GZFLc had direct activity against COX2 and correlated its anti-COX2 activity to the effect of GZFLc against induced dysmenorrhea. In addition to being effective against induced dysmenorrhea, GZFLc inhibited COX2 activity directly, indicating COX2 enzymatic assay could be used as a bioefficacy assay for GZFLc quality controls.

## 2. Materials and Methods

### 2.1. Herbal Materials and GZFL Capsules

Dried materials of Cassia Twig, Indian Bread, Peach Seed, White Peony Root, and Tree Peony Bark were purchased from Hexin Tang Raw Chinese Herbal Medicine Factory. The identities of the materials were authenticated by a botanist from Kanion Pharmaceuticals and validated by HPLC profiling by following protocols given in Chinese Pharmacopoeia (2015 Edition) or by comparison with fingerprints kept at Kanion. Representative voucher specimens were cataloged and deposited at Kanion.

**Figure 1:**
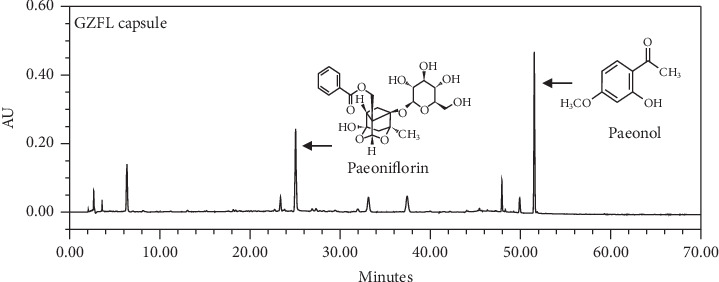
A representative HPLC chromatogram of GZFL capsule with UV detector set at 230 nm. The major components including paeoniflorin and paeonol were characterized by comparison with standards. The contents of paeoniflorin and paeonol in the batch used in this study were determined at 2.25% and 1.43%, respectively (6.97 mg and 4.43 mg in a 310 mg GZFL capsule).

GZFLc were manufactured at Kanion Pharmaceuticals using a formula essentially the same as described in Jingui Yaolue. An equal amount of Cassia Twig, Indian Bread, Peach Seed, White Peony Root, and Tree Peony Bark was used in manufacturing the capsule using a proprietary method developed at Kanion. Each capsule, weighing at 310 mg, is equal to 1.2 grams of herbal materials. The preparation was profiled by HPLC analysis (Figure 1 represents a typical HPLC histogram of GZFLc). The contents of the major components per capsule (310 mg) of the batch used for this study were determined as amygdalin at 6.22 mg, paeonol at 4.43 mg, paeoniflorin at 6.97 mg, and cinnamaldehyde at 0.60 mg.

To prepare a plant extract from individual herbs, raw materials were grounded to pass an 80-mesh screen, weighed, and mixed with distilled water (a gram of powder with 2 ml distilled water) in sealed conical tubes. The contents were extracted twice in a 95°C water bath, each time for 60 min with occasional shaking and vortexing. The extracts were pooled and then filtered through two layers of cheesecloth. After adjusting the volume to approximately 300 mg of raw material/ml (about 30 mg raw material/mouse), freshly prepared extracts were tested for their antidysmenorrhea activity (as summarized in Table 1).

### 2.2. Chemicals and Reagents

Acetonitrile of HPLC grade was purchased from TEDIA (Fairfield, OH), and analytical reagent grade trifluoroacetic acid was purchased from Nanjing Chemical Reagent Company (Nanjing, China). Ultrapure water was prepared in the lab using a Millipore water purification system (Milford, MA). Paeoniflorin (PubChem ID 442534, purity >98%) and amygdalin (PubChem ID 656516, purity >98%) were purchased from MedChem Express, and paeonol (PubChem ID 24895579, purity >99%) and cinnamaldehyde (PubChem ID 24900954, purity >95%) were purchased from Sigma Aldrich (Shanghai, China).

Antibodies against COX2 and *β*-actin were purchased from Santa Cruz. HRP-conjugated secondary antibodies were purchased from Sigma Aldrich. The ECL reagent kit was purchased from Thermo Fisher. A COX2 inhibitor screening kit was purchased from Beyotime Technologies (#S0168, Nantong, China).

### 2.3. HPLC Chromatography

HPLC studies were carried out using a Waters 6890 liquid chromatograph system. The separation was carried out on a Kromasil reverse phase C18 column (Kromasil KR100-5-C18, 4.6 *∗* 250, 5 *μ*m) using a gradient consisting of 0.02% (v/v) trifluoroacetic acid in water (TFA) as mobile phase A and acetonitrile as mobile phase B. A linear gradient program was set as follows: 0–5 min, 95% A, 5–20 min, 95–83% A, 20–30 min, 83–81% A, 30–40 min, 81–74% A, 40–60 min, 74–12% A, and 60–70 min of 12% A. The column temperature was maintained at 30°C, while the flow rate was kept constant at 1.0 ml per min.

To profile the chemicals in the plants, grounded materials were accurately weighed and extracted in a 37°C water bath with methanol and analyzed. The identity of the major components was determined by comparison with corresponding standards (as summarized in Table 1).

### 2.4. Animals and Animal Studies

The animal studies were approved by the Institutional Animal Care and Use Committee (IACUC) of Medical School of Nanjing University and by the IACUC of Kanion Pharmaceutical Co., respectively. The animal care and experimental procedures were carried out in accordance with the Guidelines for the Care and Use of Laboratory Animals set by the US NIH and Regulations for the Care and Use of Laboratory Animals (2008) set by Jiangsu Province of China.

Female ICR mice (6–8 weeks old) were purchased from Nanjing University model animal facility. The animals were housed under standard laboratory conditions, and food and tap water were provided ad libitum. For animal studies, ibuprofen or the contents (30 mg/ml) of GZFLc were resuspended in a PBS buffer containing 10% DMSO and 2% Tween 80 and administrated without storage by oral gavage.

We used an induced-dysmenorrhea model essentially as described [16]. Briefly, ICR mice were treated with saline or with estradiol benzoate (1 mg/kg/day) by i.p. route for three consecutive days. On the fourth day, the mice were randomly grouped (*n* = 5 or 10) and were treated by oral gavage with saline (*n* = 5 for untreated control; *n* = 10 for oxytocin-induced, but untreated), or with ibuprofen (50 mg/kg, *n* = 10), or a test sample (*n* = 10) at dosages as indicated. The animals were then injected with 50 U/kg of oxytocin (MedChem Express, Shanghai) by i.p. injection 30 min after drug administration. Oxytocin injection resulted in immediate writhing responses, which mainly consist of abdominal wall contractions and pelvic rotation, followed by hind limb stretches. The responses were recorded for 30 min.

We adapted a rotarod method to determine the therapeutic effect by measuring the time of a mouse staying on a rotating rod [17, 18]. After administration of oxytocin, mock- or drug-treated mice, five in a group (*n* = 5), were put on a rotarod apparatus (model YLS-4C, Zhongshidi Technologies, Beijing) with the rotating rate set at 20–30 rpm. The time that a mouse dropped off from the rotating rod was recorded and used to measure the severity of dysmenorrhea.

### 2.5. Histology Studies

For histological or immune blotting studies, the animals were sacriﬁced by cervical dislocation 20 min after oxytocin treatment. The uterine tissues were collected and fixed in 10% buffered formalin or processed for protein extraction and immune blotting studies. Fixed organs were dehydrated with a graded ethanol series and embedded in paraffin sectioning and prepared for hematoxylin and eosin staining. The slices were examined under a light microscope (Olympus BX53 microscope). The images were captured with the equipped camera and preinstalled software.

### 2.6. Western Blotting

The uterine tissues taken after writhing study were used to prepare total protein extract. Tissues, two from each group, were lysed with a lysis buffer containing 1% NP-40 and 0.1% SDS and a cocktail of protease inhibitors. The samples were left on ice for 30 min with occasional vortexing. The tissue lysates were collected after centrifugation at 10,000  ×g for 10 min at 4°C. After determining protein concentrations using a BCA method, the proteins were separated by SDS and immune-blotted with an appropriate antibody and an ECL reagent kit. The images were captured on Chem Scope equipment.

### 2.7. COX2 Activity Assay

The effect of GZFLc or pure chemicals on COX2 activity was measured using cyclooxygenase-2 inhibitor screening kit (#S0168). The kit measures the conversion of arachidonic acid to prostaglandin G2 by COX2. Briefly, recombinant human COX2 in 96-well plates was incubated at 37°C for 15 min with series-diluted GZFLc or a test compound in duplicate. COX2 specific inhibitor celecoxib was included as a positive control. The reaction mixtures were then allowed to react with arachidonic acid following the manufacturer's instruction. COX2 activity was determined by measuring the production of the final product with a fluorescent probe. The excitation wavelength was set at 560 nm and the emission wavelength was at 590 nm. The inhibitory effect on COX2 was calculated using the following equation: percentage of inhibition = (1 − average net inhibitor fluorescence/average net noninhibited fluorescence) × 100%.

### 2.8. Statistical Analysis

The data were expressed as average ± SE or mean ± SD. One-way ANOVA was used for comparison of statistic significances among paired groups.

## 3. Results

### 3.1. Evaluation of GZFLc Antidysmenorrhea Effect Using an Oxytocin-Induced Murine Model

We and others have shown that GZFLc or similar formulae were able to reduce pains and ameliorate symptoms associated with dysmenorrhea. To delineate the major ingredients responsible for GZFLc effect, the contents of GZFLc were resuspended in PBS and tested in a mouse dysmenorrhea model developed by Yang et al. [16]. In this regard, forty-five ICR female mice were treated with estradiol for three consecutive days. On the fourth day, the animals were randomly grouped into five groups (Table 2) and were treated with ibuprofen (50 mg/kg, orally) or with GZFLc at 150 and 300 mg/kg, respectively. We chose those two dosages since they fall within the recommended ranges for human usage. Thirty minutes after the treatment, the animals were injected by i.p. route with oxytocin at 50 U/kg or with saline for the mice in the control group. Oxytocin injection induced significant but momentary pain in mice that lasted about 20 min, including signs of abdominal wall contractions, pelvic rotation, and rear limb stretches. As shown in Table 2, mice treated with ibuprofen had significantly less symptoms of writhing responses. For comparison, GZFLc at 150 and 300 mg/kg markedly reduced the frequency of pain responses, particularly those of pelvic twisting and rear limb stretches, from 20 times to 7 and 4 times, respectively, indicating that GZFLc was effective against oxytocin-induced writhing responses. A comparison between the two groups was made. As shown in Table 3, except that there was no difference between the control group and ibuprofen group, there was a significant difference between the other two groups (*p* < 0.05).

**Figure 2:**
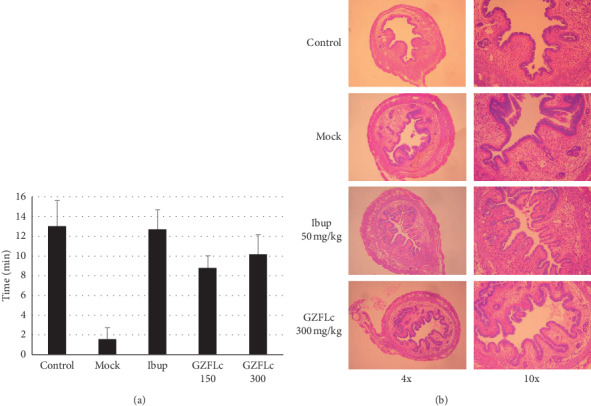
Effect of GZFLc on motor coordination and uterus inflammation. (a) GZFLc effect on motor coordination and balance of mice undergoing induced dysmenorrhea. ICR female mice were treated with estradiol for three consecutive days. The mice were randomly grouped (*n* = 5) and were treated with saline, ibuprofen, or GZFLc 30 min prior to being subjected to a rotarod assay. The time of a mouse staying on a rotating rod was recorded and used as a measure of GZFLc effect on induced dysmenorrhea. The experiment was performed two times independently. (b) ICR female mice were treated with estradiol for three consecutive days. On the fourth day, the animals (*n* = 3) were treated with ibuprofen or GZFLc 30 min prior to oxytocin injection. Twenty minutes later, the animals were killed, and the uteruses were removed and fixed in 10% formalin for histology study. Sections of 5 *μ* in thickness were stained with hematoxylin and eosin (H & E). The slices were examined and photographed at 4x and 10x magnifications. Con, noninduced untreated controls; Mock, oxytocin-induced and vehicle-treated; Ibup, ibuprofen at 50 mg/kg; GZFLc 150 and 300 mg/kg, respectively.

Dysmenorrhea causes pain that might affect motor coordination and balance of the animals. We attempted to use a rotarod assay to quantitatively measure the effect of GZFLc against dysmenorrhea. In this regard, randomly grouped mice (*n* = 5) were subjected to a test by putting the animals on a rotating bar. The time that an animal stays on the rotating bar was recorded and used to measure the effect of ibuprofen or GZFLc treatment. As shown in Figure 2(a), the average time of mice hanging on the bar was recorded as 12.95 ± 2.68 min, while the time dropped to less than 2 min in the oxytocin-induced group. Treatment with ibuprofen brought the hanging time close to that of the nonoxytocin-induced controls (12.63 ± 2.07 min). Treatment with 150 and 300 mg/kg GZFLc resulted in the hanging time to be 8.74 and 10.12 min, respectively, indicating that GZFLc treatment alleviated the discomfort of mice suffering from dysmenorrhea.

The antidysmenorrhea effect was further investigated by examination for signs of proinflammatory responses. In this study, mice were killed 20 min after oxytocin treatment. The uterine tissues were removed and fixed for H & E staining (Figure 2(b)). Compared with the uteruses from the control group (Con), the lamina propria had severe edema and the basalis layer of endometrium was thicker, while the endometrium had more endometrial glands in estradiol-treated and oxytocin-induced group (Mock), indicating that the animals were in the estrus stage. The lamina propria in ibuprofen (Ibup) and in GZFL-treated groups had markedly reduced edema compared with oxytocin-induced and vehicle-treated controls (Mock group), while no obvious differences were noticed in the basalis layers and the endometrium among Mock and GZFLc-treated groups.

The results together demonstrated that GZFLc treatment was effective against induced dysmenorrhea.

### 3.2. GZFLc Inhibits COX2 Activity in an Enzymatic Assay

**Figure 3:**
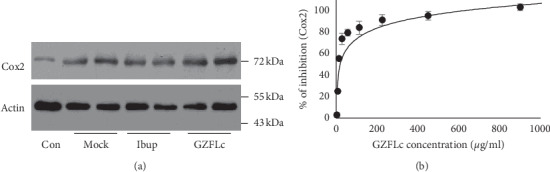
GZFLc effect on COX2 expression and COX2 activity. (a) GZFLc treatment effect on COX2 expression. Uterus tissues were removed from mock-treated or drug-treated samples (*n* = 2). The expression of COX2 was detected by immunoblotting analysis using *β*-actin as a loading control. COX2 expression was upregulated in estradiol and oxytocin-treated samples. Treatment with ibuprofen at 50 mg/kg or GZFLc at 300 mg/kg for 30 min marginally affected COX2 expression. The experiments were performed twice independently. (b) Determination of GZFLc effect on COX2 activity with an in vitro enzyme assay. The content in the GZFLc was weighed and extracted with distilled water (1.8 mg/ml) at 37°C for 2 hr. The liquid was collected by centrifugation and cleansed by filtering through a 0.2 *μ* filter. After serial dilution, the liquid was tested for its inhibitory activity against COX2 activity. Celecoxib was included as a positive control. Data are presented as average ± SE of duplicated samples.

Prostaglandin F2*α* production has been identified as a critical factor for primary dysmenorrhea [6, 10, 19]. We therefore examined whether GZFLc treatment affected COX2 expression, a rate determining enzyme that catalyzes arachidonic acid conversion to prostaglandins. The mouse uterus tissues from untreated or ibuprofen or GZFLc-treated samples were used for COX2 expression studies. As shown in Figure 3(a), COX2 expression was relatively low in the untreated samples, while its expression was significantly upregulated in estradiol and oxytocin-treated samples. Unlike results from those with prolonged treatment, we detected no significant alteration of COX2 expression in GZFLc or in ibuprofen-treated samples, indicating GZFLc treatment did not change COX2 expression.

### 3.3. Evaluation of Individual Herbal Extracts Effects on Mouse Dysmenorrhea Model

**Figure 4:**
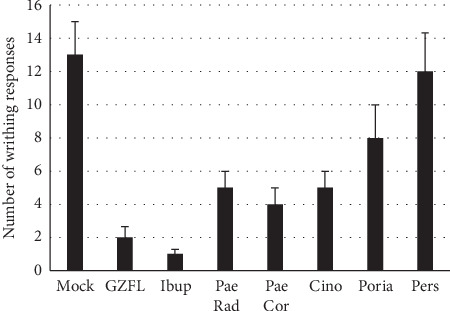
Inhibition of writhing responses by herbal extracts. Aqueous extracts were reformulated to match relative contents in the GZFL capsule and tested in oxytocin-induced dysmenorrhea model. Writhing responses were recorded and plotted as mean ± SD (*n* = 5). PaeRad, Paeonia radices; PaeCor, Paeonia cortexes; Cinno, *Cinnamomum cassia* twigs; Poria, *Poria cocos*; Pers, Semen Persicae.

To preliminarily determine the contributions of individual components, grounded plant materials were extracted at 95°C for 60 min in sealed tubes. The extracts were cleansed by filtering through two layers of cheesecloth and were reconstituted to match the contents in GZFL formula. The extracts were then tested individually on the oxytocin-induced dysmenorrhea model. Although the effect of individual extracts was less potent compared to that of 300 mg/kg of GZFLc, the extracts from radices Cassia Twig, White Peony Root, and Tree Peony Bark, but not Indian Bread and Peach Seed, showed activities in reducing writhing responses (Figure 4). In general, extracts from Cassia Twig, White Peony Root, and Tree Peony Bark reduced the frequencies of pelvic twisting and limb stretches by approximately 60–70%, indicating that those materials were partially responsible for the antidysmenorrhea activity of GZFLc.

### 3.4. Correlation Analysis of Major Components to the Antidysmenorrhea Effect of GZFLc

**Figure 5:**
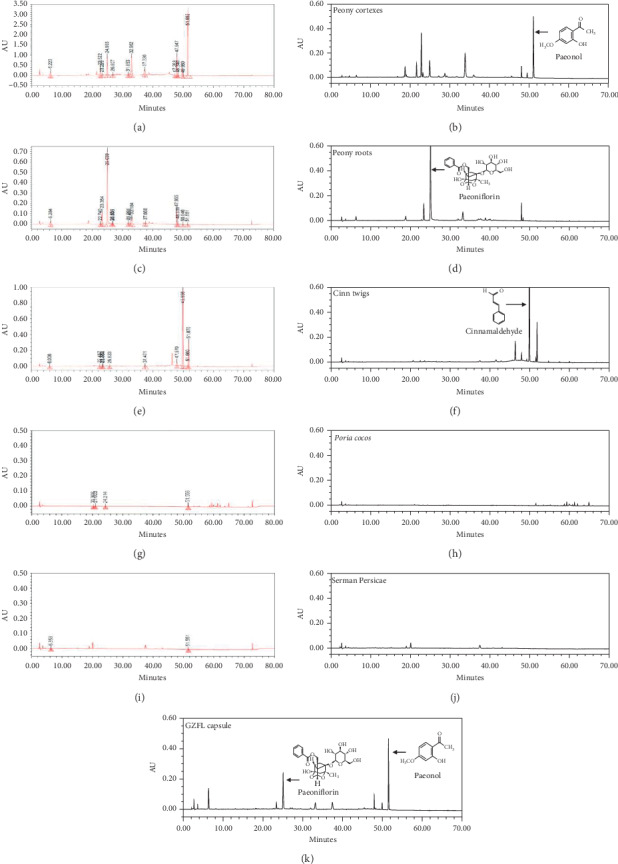
HPLC profiling of the methanolic extracts of plant materials. Grounded plants were extracted with methanol. The extracts were profiled by HPLC analysis with an UV detector set at 230 nm.

**Figure 6:**
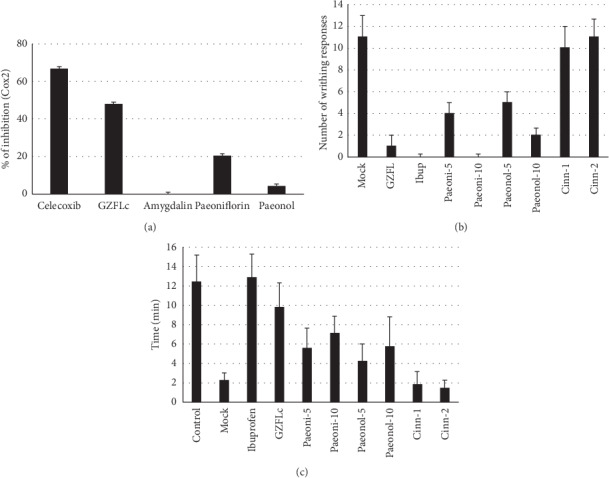
Effect of paeoniflorin, paeonol, and cinnamaldehyde on COX2 activity and against induced dysmenorrhea. (a) The major components in GZFLc were paeoniflorin, paeonol, and cinnamaldehyde (refer to Section 2). Their effect against COX2 activity was determined using an enzyme-based assay. (b) Paeoniflorin, paeonol, and cinnamaldehyde were tested against induced dysmenorrhea at the following dosages: paeoniflorin at 5 and 10 mg/kg, paeonol at 5 and 10 mg/kg, and cinnamaldehyde at 1 and 2 mg/kg. The dosages were selected since they were close to the amounts in GZFLc at 300 mg/kg (refer to Section 2). GZFLc at 300 mg/kg and ibuprofen at 50 mg/kg were included as controls. Both writhing responses and the time of mice staying on the rotating bar were recorded. The experiment was performed twice independently.

Based on the results from activity-directed assay, we investigated whether the main components were representative of GZFLc activity. The chemical contents in GZFLc have previously been identified, with cinnamaldehyde, paeoniflorin, paeonol, and amygdalin as major components [20, 21]. We therefore profiled the chemicals from Cassia Twig, White Peony Root, and Tree Peony Bark by HPLC analysis since extracts from those plants showed activity against oxytocin-induced writhing responses. Consistent with previous studies, HPLC profiling indeed showed paeoniflorin, paeonol, and cinnamaldehyde as the major representative compounds in those plants (Figure 5). We therefore tested whether those major components represented GZFLc activity. Paeoniflorin at 30 *μ*M partially blocked COX2 activity, while paeonol and cinnamaldehyde showed marginal to no activity in the same enzyme assay (Figure 6(a)). We also tested whether those compounds blocked oxytocin-induced dysmenorrhea. We used dosages of 5 and 10 mg/kg for paeoniflorin (0.5 mg/ml) and paeonol (0.5 mg/ml) and 1 and 2 mg/kg for cinnamaldehyde (0.1 mg/ml) in animal studies since those dosages would fall close to the ranges of the compounds in the capsule (refer to Section 2). We found that administration of paeoniflorin and paeonol, but not cinnamaldehyde, reduced the numbers of limb stretches and pelvic twisting (Figure 6(b), upper panels). The treatment also prolonged the time of mice staying on a rotating bar (Figure 6(b), lower panel). The effect was more profound for paeoniflorin and paeonol (Figure 6(b)). In addition to showing that these compounds represent GZFLc activity against oxytocin-induced dysmenorrhea, the results suggested to us that inhibition of COX2 in part represents GZFLc antidysmenorrhea activity.

## 4. Discussions

One of the major factors affecting the quality of Chinese medicinal preparations is the lack of appropriate assays that can be used for quality control and efficacy testing. Therefore, fingerprinting based on HPLC profiling of the typical/major components in individual plants or in a preparation is currently a predominant approach for quality control. Albeit powerful and being useful, this approach does not typically reflect the potency of a preparation since the active components as well as the mechanism(s) of action are in general less characterized.

In this study, we first defined the effectiveness of GZFLc using an induced-dysmenorrhea model. We showed that GZFLc at dosages relevant to clinical usages relieved dysmenorrhea symptoms and improved motor coordination of mice undergoing induced dysmenorrhea. Mice from GZFLc-treated group had significantly less edema in the lamina propria. It is not surprising that we did not detect obvious alteration in COX2 expression after a short period of treatment. Instead, we found that GZFLc targeted COX2 activity directly. This finding suggested that GZFLc exhibits its antidysmenorrhea effect likely through inhibition of COX2. This study highlights the importance of modern chemical and pharmacological research to our understanding of Chinese medicine since the identification of a mechanism of action may lead to the establishment of a more appropriate bioassay for GZFLc quality control and efficacy validation.

Our study linked paeoniflorin and paeonol, two of the major components in GZFLc, to its antidysmenorrhea activity. A bioassay-guided fractionation may lead to the identification of compounds related to GZFLc antidysmenorrhea activity. Instead, we tested the major components for their antidysmenorrhea activity and have identified both paeoniflorin and paeonol with antidysmenorrhea activity. Although the essential oil from the Cassia Twig was effective against primary dysmenorrhea in an animal model [22, 23], the dosage to accomplish the reported activity was relatively high compared to that of clinical usage. The finding from this study is consistent with results from our previous findings. Using a knock-out strategy, we deduced that fractions containing those two components were responsible for GZFLc anti-inflammatory and uterus contraction activity [20]. Although detailed studies are required to determine an optimal combination of paeonol and paeoniflorin, our study nonetheless points to a direction that the relative contents of those compounds may serve as a critical marker for GZFLc quality control.

Wenjing formulae are among the most commonly prescribed remedies for primary dysmenorrhea [3], of which several contain *Paeonia spp* [24]. Herbal medicine may be advantageous since those used for the treatment of primary dysmenorrhea tend to have analgesic effect [25]. Indeed, both the White Peony Root and Tree Peony Bark contain paeonol and paeoniflorin [26]. The reported activity of paeonol includes anti-inflammatory and analgesic effects [27]. Paeonol also inhibits anaphylactic reaction by regulating histamine and TNF-*α* production [28]. Paeoniflorin attenuates neuropathic pain via MAP-kinase pathway [29]. It also mediates neuroprotective effect, hence ameliorating the function of cholinergic nerve [30–32]. Paeoniflorin also relieves oxidative stress and downregulates NF-*κ*B expression, hence damping proinflammatory cytokine production [33]. It has been reported that prolonged administration of paeoniflorin in mice with induced colitis resulted in significant downregulation of the mRNA expression of proinflammatory mediators like MCP-1, COX2, TNF-*α*, and IL-6 [34–36], while paeonol exerts anti-inflammatory effect through inhibition of iNOS and COX2 expression and NF-*κ*B activation [27, 37, 38]. It is worth noting that paeoniflorin displayed more potent effect in a rotarod assay than in the COX2 inhibition assay. It remains to be determined whether paeoniflorin exhibits dual function by targeting both cyclooxygenase and the nociceptive pain associated with dysmenorrhea.

## Figures and Tables

**Table 1:** Content of marker constituent from five raw materials.

Raw material	Active/marker constituent chemical class	Active/marker constituent	Content (%)
Cassia twig	Volatile oil	Cinnamaldehyde	1.8
Indian bread	Terpene	Pachymic acid	0.06
Peach seed	Glycoside	Amygdalin	4.0
White peony root	Terpene	Paeoniflorin	3.0
Tree peony bark	Volatile oil	Paeonol	3.0

This table shows the compound types of main or active ingredients and the content of main indicator ingredients in the five herbs. The content refers to the ratio of the weight of marker constitutive to the original raw material.

**Table 2:** Inhibition of writhing responses by GZFLc in an oxytocin-induced dysmenorrhea model^*∗*^.

Group (*n*)	Oxytocin (i.p.)	Treatment (p.o.)	Writhing
Control (5)	−	Saline	N. S.
Mock (10)	+	Saline	14.7 ± 2.7
Ibuprofen (10)	+	Ibuprofen, 50 mg/kg	0.5 ± 0.7
GZFLc 150 (10)	+	GZFLc, 150 mg/kg	6.0 ± 1.9
GZFLc 300 (10)	+	GZFLc, 300 mg/kg	3.3 ± 1.3

^*∗*^ICR female mice were treated with estradiol for three consecutive days. The mice were randomly grouped into five groups. Thirty minutes prior to oxytocin injection, the mice were treated with normal saline, with ibuprofen, or with GZFLc at 150 and 300 mg/kg by oral gavage. To induce dysmenorrhea, mice were injected with oxytocin or with saline by i.p. route. The frequencies of pelvic twisting and rear limb stretching were recorded for 30 min and plotted as a measure of writhing responses (mean ± SD). The experiment was performed two times independently.

**Table 3:** The result of pairwise of comparison.

Group	Mean difference	95% CI of mean difference		Statistically significant
Mock-ctrl	14.7000	11.5777	17.8223	^*∗∗∗*^
Ibuprofen-ctrl	0.5000	−2.6223	9.1223	
Mock-ibuprofen	14.2000	11.6506	16.7497	^*∗∗∗*^
Mock-GZFLc 300	11.4000	8.8506	13.9494	^*∗∗∗*^
Mock-GZFLc 150	8.7000	6.1506	11.2494	^*∗∗∗*^
GZFLc 300-ibuprofen	2.8000	0.2506	5.3494	^*∗∗∗*^
GZFLc 150-GZFLc 300	2.7000	0.1506	5.2494	^*∗∗∗*^

Comparisons significant at the 0.05 level are indicated by ^*∗∗∗*^.

## Data Availability

The datasets used and analyzed during the current study are available through sending an email to the corresponding author.
